# Deep-Water Renewal Events; Insights into Deep Water Sediment Transport Mechanisms

**DOI:** 10.1038/s41598-020-63123-3

**Published:** 2020-04-09

**Authors:** K. Ayranci, S. E. Dashtgard

**Affiliations:** 1 College of Petroleum Engineering & Geosciences, King Fahd University of Petroleum & Minerals, Dhahran, 31261 Saudi Arabia; 2 Applied Research in Ichnology and Sedimentology (ARISE) Group, Department of Earth Sciences, Simon Fraser University, 8888 University Drive, Burnaby, British Columbia, V5A 1S6 Canada

**Keywords:** Physical oceanography, Ocean sciences

## Abstract

Deep-water renewal (DWR) events are characterized in the Strait of Georgia, Canada using 11 years of real-time physical and chemical oceanographic data and seafloor videos. At least 6 DWRs occur per year at 300 m water depth and each event continues for over 3 days. They initiate during neap tides and are associated with increased turbidity. In the spring, DWRs introduce cold, oxygenated and nutrient-poor waters, and in the fall they introduce warm, oxygen-depleted, nutrient-rich and saline waters. Although the timing and magnitude of DWRs differ from year to year, we demonstrate that they are not restricted to two seasons, but continue throughout the year. High-resolution videos of DWRs show that these events comprise a plume of high suspended sediment concentration that flows parallel to the basin axis and deposits approximately 1.5 cm per event.

## Introduction

Deep-water currents are efficient sediment transport mechanisms in modern and ancient systems^1–6^, yet there is little known about their physical and chemical oceanographic properties (see exceptions^2,7,8^), and even less about the physical manifestations of these currents. In the Strait of Georgia (SoG), Canada, deep-water renewal (DWR) events are documented as deep inflow currents^7,9^ that cause fluctuations in chemical oceanographic properties such as salinity, dissolved oxygen, temperature and nutrient content^7,10–12^. In the spring, DWRs introduce cold, oxygenated and nutrient-poor waters into the SoG, and in the fall they introduce warmer, oxygen-depleted, nutrient-rich and saline waters^7,9,13^.

**Figure 1:**
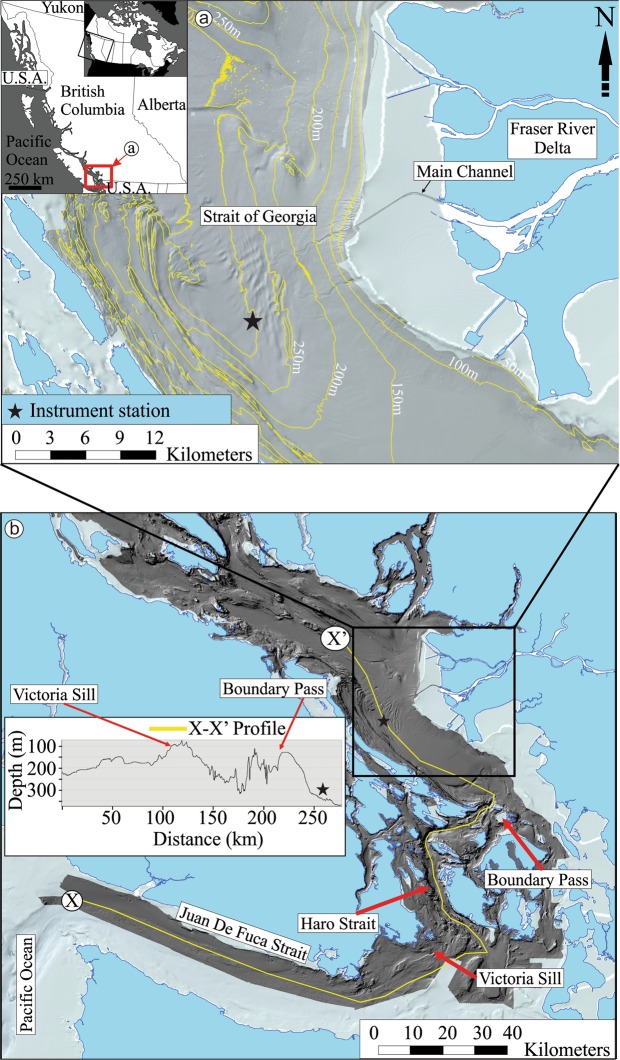
Location map. (**a**) Bathymetric map of the Strait of Georgia off the Fraser River Delta and the position of the instrument station (black star). The inset map shows the position of a (red box) in Canada and British Columbia. (**b**) Bathymetric map of the central and southern Strait of Georgia. The inset graph represents the bathymetric profile between the Juan de Fuca Strait to the central SoG (X-X’). Maps and bathymetric profile image were created using ArcGIS software (version: 10.7.1; www.arcgis.com).

The drivers of DWRs in the SoG are multiple and include Pacific Ocean upwelling, Fraser River discharge, and climate conditions^7,10^. The multiple controlling mechanisms makes it difficult to accurately predict the character of these events, which varies from year to year. Regardless of the driving mechanisms, DWRs are associated with intrusion of dense Pacific Ocean waters into the SoG through the narrow Juan de Fuca Strait (Fig. 1). The dense Pacific waters are trapped and concentrated at the Haro Strait by sills located at Boundary Pass. The trapped dense waters flow fortnightly into the SoG during neap tides when there is minimum tidal mixing at the sills causing strong water stratification^7,9,10,14^. Dense waters are then pushed into the SoG by northward-flowing flood tides.

In this study, we analyze all available real-time physical and chemical oceanographic data (2009–2019) recorded at 300 m water depth off the Fraser River Delta and in the SoG (Fig. 1). In addition, we present still images extracted from video footage of a DWR in the SoG and discuss the physical manifestations of its passage.

## Results

### Study region

The SoG is situated between Vancouver Island and the mainland of British Columbia, Canada (Fig. 1). Hydrodynamic conditions are mainly controlled by a combination of factors, including Pacific Ocean upwelling, strong tidal currents, climatic variations and discharge from the Fraser River^7,13^. The Fraser River, which is the largest river on the west coast of Canada, discharges into the SoG and takes the form of a hypopycnal plume, the latter of which is commonly deflected to the south-southwest by wind-driven waves^15–17^. Along the SoG, northward flowing flood tides are typically stronger than southward flowing ebb tides; thus, the net sediment transport direction is to the north^13,16,18^.

### DWRs from 2009 to 2019

**Figure 2:**
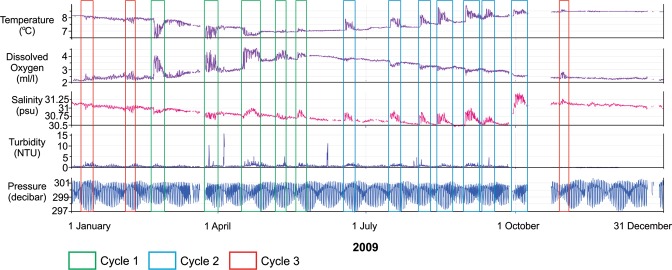
Oceanographic measurements from the central Strait of Georgia (300 m water depth) in 2009 and during multiple DWRs. Each DWR (indicated by the rectangles and color-coded to cycles) starts at the neap tidal cycle. The character of DWRs varies significantly between cycles, particularly between cycles 1 and 2, whereas the vast majority of the DWRs in each cycle show consistent characteristics across all years. The timing of DWRs varies significantly probably due to the mechanisms controlling dense Pacific Ocean intrusion. For the complete data set refer to Supplementary Fig. S1.

One-hundred and thirty-six DWRs were recorded between January 2009 and October 2019 (Supplementary Fig. S1). Although chemical characteristics of DWRs vary throughout the year, all events started during neap tidal cycles and last for at least 3 days. Given that the dataset is extensive, we illustrated oceanic properties of DWRs in 2009 which comprises one of the most complete datasets of the eleven years of data (Fig. 2). Other years are included as supplementary data (Supplementary Fig. S1).

Variations in oceanographic parameters throughout the year render it challenging to compare individual DWRs both intra- and inter-annually. In particular, discharge from the Fraser River, variations in tidal currents, and climate conditions over the Pacific Ocean, and even living organisms (e.g., consuming excessive oxygen during certain times of the year) all appear to impact the timing and characteristics of DWRs^7,9,12,19^. Although there are some annual variations in the timing of these events, when eleven years of data are compared, generalities emerge in the character of these events (Supplementary Fig. S1). We divide DWRs into three cycles based on their chemical characteristics. These cycles occur at different times in the year and the start and/or end of each cycle may vary by up to one month from year-to-year (Supplementary Fig. S1). When eleven years of data considered, the first cycle (C1) consistently occurs between February and July, C2 occurs between June and November, and C3 occurs between October and March (Supplementary Fig. S1).

### Physical and chemical characteristics of DWRs by cycle

In 2009, C1 occured between February and May, during which time the amplitudes of fluctuations in oceanographic properties during DWRs were pronounced. Thirty-nine DWRs were recorded in C1 between 2009 and 2019, 5 of which were recorded in 2009. In general, temperature during C1 DWRs decreased, although some DWRs towards the end of C1 exhibited a gradual shift from a decreasing to a slight increasing pattern (e.g., 2009 and 2012; C1 in Fig. 2 and Supplementary Fig. S1). Dissolved Oxygen (DO) shows a consistently increasing pattern for all DWRs in C1 while salinity does not show a well-defined trend with the exception of a generally increasing pattern during DWRs in the second half of C1 (Fig. 2). In 2009, the average temperature change during DWRs was −0.53 °C (standard deviation (stdev): 0.8), average salinity variation was +0.02 psu (stdev: 0.2), and average DO variation was +1.30 ml l^−1^ (stdev: 0.6). Turbidity also increased by an average of 6.56 Nephelometric Turbidity Units (NTU; Supplementary Table S1) (stdev: 11.4).

The second cycle (C2) extends from approximately June to October, in 2009. Fifty DWRs were recorded in C2 between 2009 and 2019, and 7 of these occurred in 2009. The chemical and physical characteristics of DWRs in C2 are consistent and of greater amplitude compared to those during other cycles. Salinity values show the most striking increasing trends with an average increase of 0.44 psu (stdev: 0.2) during events in 2009 (Fig. 2). Temperature typically shows a sharp increase at the beginning of C2 DWRs and slowly returns to ambient temperatures throughout the cycle. In 2009, the average temperature increase during C2 DWRs was 0.91 °C (stdev: 0.3). Dissolved oxygen generally decreases during C2 DWRs, and in 2009, the average DO decrease was 0.43 ml l^−1^ (stdev: 0.1). Turbidity during 2009 C2 DWRs increased on average by 6.56 NTU (Supplementary Table S1) (stdev: 5.7).

The third cycle (C3) extends from October to February, in 2009. Forty-seven DWRs were observed in C3 between 2009 and 2019 and three of these were in 2009. DWRs in C3 are characterized by weak signatures and they show noticeable variations between years (Fig. 2 and Supplementary Fig. S1). Salinity and temperature variations are represented by increasing patterns, but in some years are represented by weak decreasing patterns (Supplementary Fig. S1). DO shows an increasing pattern through events. The C3 DWRs in 2009 display an average temperature decrease of 0.18 °C (stdev: 0.3), salinity decrease of 0.05 psu (stdev: 0.2), and a DO increase of 0.54 ml l^−1^.(stdev: 0.1). Turbidity suspiciously showed no variation during 2009 DWRs in the first half of C3. In the second half of C3, turbidity increases by an average of 2.14 NTU (Supplementary Table S1) (stdev: 0.7).

### Character of a DWR: the DWR0312 event

**Figure 3:**
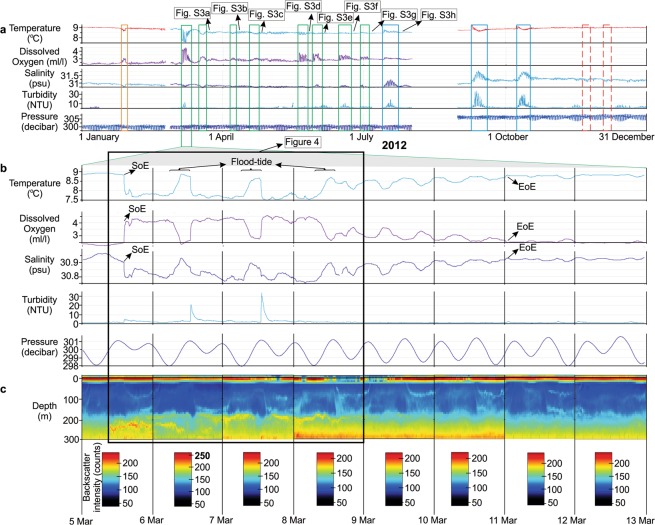
Oceanographic measurements from the central Strait of Georgia (300 m water depth) during the DWR0312 event that occurred between March 5 and 11, 2012. (**a**) Measurements for all of 2012 with DWRs indicated by the green boxes. The timing of photos in Fig. 5 are indicated by the arrows. (**b**) Daily changes in seawater parameters during the DWR0312 event. (**c**) Acoustic backscatter intensity (counts) from the ADCP showing increased bottom water turbidity. Each day has its own ADCP color legend due to a significant increase in turbidity on March 6, 2012. SoE: start of event; EoE: end of event.

**Figure 4:**
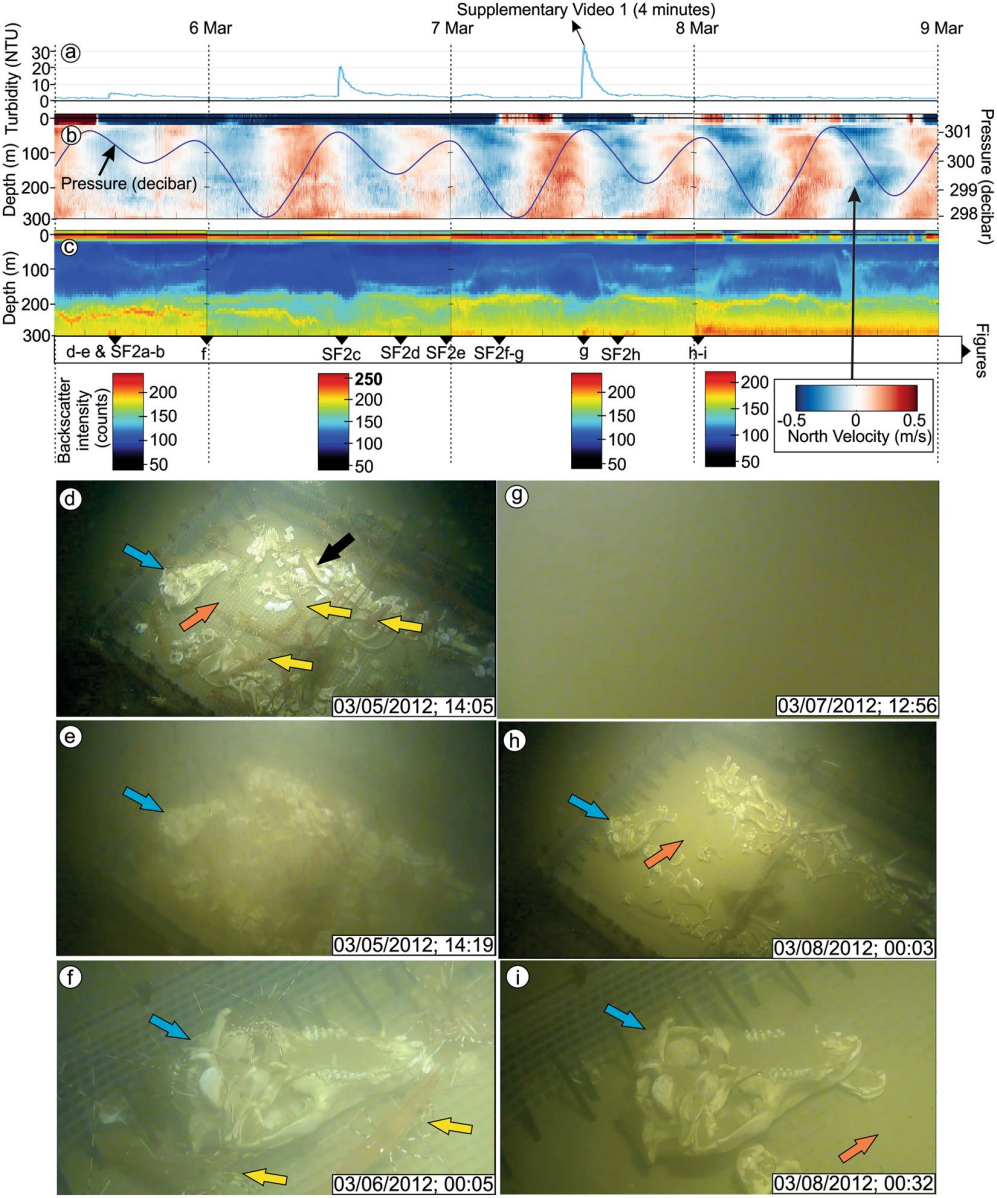
Physical and chemical variations and still images captured from video footage of the DWR0312 event. (**a–c**) Variations in physical oceanographic properties during the DWR0312 event. The black triangles at the bottom of **c** indicate the timing of video-image capture (note that “S” indicates Supplementary Figure). (**d–i**) Video image captures from before and during the DWR0312 event. In all images, the blue arrow indicates the pig skull, the orange arrow indicates the mesh bottom of the cage and the yellow arrows point to shrimp. Please note that (**f**,**i**) are close up images of the skull that are located close to the edge of the cage.

Video footage of a DWR was acquired in 2012 and during a C1 event (Fig. 3a–c). This particular DWR started on March 05, 2012 at approximately 14:08 pm and at the end of a flood tide (beginning of an ebb tide). The DWR lasted approximately 10.5 days, ending on March 11, 2012 (Fig. 3b). Below are descriptions of the changes in physical and chemical signatures (Figs. 3b and 4a), and acoustic doppler current profiles (ADCP; Figs. 3c and 4b,c) during this DWR. Video footage also captured the event at multiple times throughout it (Fig. 4d–i, Supplementary Fig. S2, and Supplementary Video S1). We call this DWR “DWR0312”.

### Chemical and physical characteristics of the DWR0312 event

The DWR0312 event started with a sharp decrease in both temperature and salinity, and a sharp increase in DO (Fig. 3b). Temperature dropped from 8.87 °C to 7.78 °C (−1.09 °C) and, salinity decreased from 30.91 to 30.80 psu (−0.10 psu). Conversely, DO sharply increased from 2.44 to 4.10 ml l^−1^ (+1.66 ml l^−1^). Both temperature and salinity remained depleted throughout the DWR reaching minima of 7.48 °C and 30.75 psu, respectively. DO remained high through most of the event reaching a maximum of 4.53 ml l^−1^ and this coincided with minimum salinity (Fig. 3b).

The onset of the DWR0312 event corresponds to the flood tide, although it reaches the instrument array during the ebb tide (Figs. 3b and 4b). All the changes in chemical oceanographic properties during the DWR0312 event temporarily return to their ambient values during flood tidal currents for 5–7 hours each time (Figs. 3b and 4b). In the first three days, these trends are consistent while in the following seven days the chemical properties gradually reset back to ambient values (Fig. 3b).

The DWR0312 event started with an increase in turbidity from 1.58 to 4.82 NTU (+3.24 NTU) followed by two more significant increases coinciding with the end of strong flood tides (Fig. 4a). Turbidity reached its peak of 29.69 NTU following the second flood tide, and after which it returned to ambient values and remained low for the rest of the event.

Backscatter intensity and current velocity data, measured by ADCP provide insight into the hydrodynamics within the 300 m water column during the DWR0312 event (Fig. 4b,c). When the DWR0312 event initiated, a slight increase in particle intensity occurred (Fig. 4a,c) and northward directed flow velocity reached approximately 0.5 m s^−1^ (Fig. 4b). Two major and strong pulses appear in the following two days matching with increases in particle intensity at the end of strong flood tides (Fig. 4a–c). Following these two pulses bottom water particle intensity reduces and returns to ambient conditions.

### Video footage of the DWR0312 event

**Figure 5:**
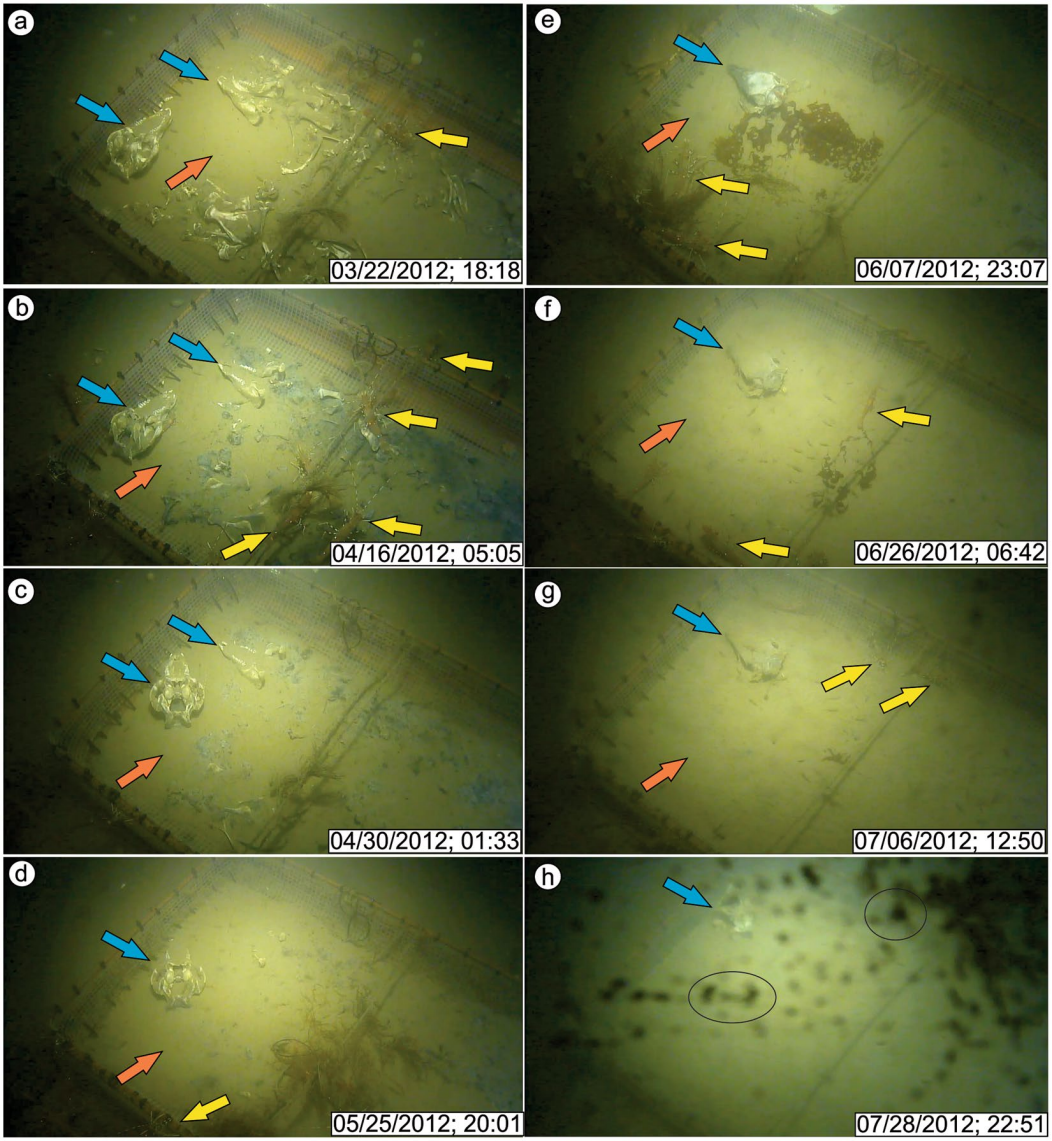
Video captures showing bottom waters after each DWR following the DWR0312 event. (a-g) Still images representing turbidity-cleared bottom waters following DWRs. Bones, skulls (blue arrows), meshed cage bottom (orange arrow) and shrimps (yellow arrows) are visible. Note that bones, skulls, and the meshed cage bottom show progressive sediment accumulation following each DWRs. Refer to Fig. 3 for timing of these events. h) This image represents one of the last images captured by the camera in 2012. Following this period, camera was recovered probably due to the biofouling (black circles).

During the DWR0312 event, the camera recorded a clean image of the seafloor, including exposed pig bones and epifaunal shrimp (Figs. 4 and 5, Supplementary Fig. S2, and Supplementary Video S1). In order to describe the DWR0312 event, we analyzed all available video data between March 1 and 16, 2012. Five minutes before the event initiated, bottom waters showed no signs of increased suspended sediments (i.e., turbidity) and a large number of shrimp were present (Fig. 4d). Video recording was paused three minutes before the DWR0312 event so the onset of the DWR was not recorded. The next video started 8 minutes after initiation of the event at which time visibility was significantly reduced due to increased turbidity (Fig. 4e).

Two hours from the initiation of the DWR0312 event, the bones were still partially visible, and sediment began to accumulate on the bones (Supplementary Fig. S2a). Approximately 2.5 hours after initiation of the event, visibility in the water column improved in response to reduced turbidity (Supplementary Fig. S2b), and epifaunal shrimp feeding on the pig carcasses remained during this stage (Supplementary Fig. S2b). Nine hours after initiation, the skull showed more accumulation of sediments (Fig. 4f). The water column remained relatively free of turbidity until approximately 12:40 pm on March 6, 2012. At this time the first of two major turbidity peaks were recorded and following which, visibility was reduced to zero (Supplementary Fig. S2c). Visibility returned at approximately 8:00 pm, on March 6 (Supplementary Fig. S2d) as turbidity in the water column subsided (Fig. 4a). Following the first major turbidity peak, more sediment had accumulated on the pig skull compared to the first slight turbidity increase (Fig. 5f and Supplementary Fig. S2e).

The second and last turbidity peak occurred on March 7, 2012 at 12:55 pm (Fig. 4a), and in under 30 seconds visibility was reduced to zero (Fig. 4g and Supplementary Video S1). Two hours after the second peak (at 3:57 pm) visibility returned and most bones were partially buried, the meshed bottom was covered, and all shrimp had left the area (Supplementary Fig. S2h). Shrimp returned to the area 3 hours later, but in low numbers. Eleven hours following initiation of the second turbidity peak, approximately 1.5 cm of sediment had accumulated on top of the skull and around the meshed-cage bottom (Fig. 4h,i). After 2 days the population of shrimp reached high levels, but did not reach the same density as before the event.

In order to compare sedimentation rates during DWRs, all available video footage from 2012 was analyzed and similar increased turbidity was recorded during other DWRs (Figs. 3–5). Eight DWRs (7 in C1, 1 in C2) occurred after the DWR0312 event (March 5, 2012) and before the removal of the camera (August 1, 2012). Each DWR deposited sediments and contributed to the burial of the bones (Fig. 5a–h). Although it is not possible to precisely and directly measure the sedimentation rate in each event due to both the lack of vertical scale in the video footage and to organism disturbance (e.g., shrimp or octopus; Supplementary Fig. S2g) of bones, we approximated the thickness of the humerus bone (black arrow, Fig. 4d) as 6 cm using the bottom mesh size (1 cm) as a scale. The humerus bone was completely buried following three DWRs after the DWR0312 event, indicating that each DWR deposited approximately 1.5 cm of sediment.

## Discussion

Regardless of the controlling factors, dense Pacific Ocean waters penetrate into the Juan de Fuca Strait, especially during upwelling periods, and get trapped by sills at the Boundary Pass before entering into the central SoG (Fig. 1;^9^). During spring tides, strong mixing occurs at the Boundary Pass preventing the dense waters from passing the sills, thus concentrating them at the bottom of the Haro Strait (Supplementary Fig. S3). The mixing probably causes resuspension of settled sediments at the sills increasing the turbidity of dense waters as well. During neap tides, weaker tidal currents cause less mixing and results in strong stratification of fresh surface waters and dense bottom waters (Supplementary Fig. S3). Following strong flood tidal currents, these dense waters are pushed into the central SoG changing physical and chemical characteristics of bottom waters for 3 days or longer (Supplementary Fig. S3;^7,9,13^).

### Chemical characteristics of DWRs

Of the three cycles of DWRs described in this study, C1 and C2 show the most pronounced and consistent chemical variations (Fig. 2 and Supplementary Fig. S1), and DWRs in these two cycles (spring and fall) are identical to those described previously^7,9,13^. Conversely, DWRs in C3 are difficult to recognize^20^ mainly because chemical signatures are weak or absent.

We present three hypotheses for the weak or absent signatures during C3. (1) Intrusion of Pacific Ocean water into the SoG is either very limited or not present during these cycles. (2) During C3, DWRs do not reach the central SoG largely because the flows are weak^21^. (3) The chemical characteristics of DWRs and SoG bottom waters are similar during C3, and hence, DWRs cannot be easily discerned. 1) The first hypothesis is plausible and may be explained by climate conditions. For example, seasonal changes in major wind directions may affect dense water intrusions causing a lack of DWRs at certain times of the year as is the case in some fjords (e.g., the Gullmar Fjord and Norwegian fjords^22,23^. 2) The second hypothesis can play a role, particularly when there is strong mixing causing weak water column stratification. 3) The third hypothesis seems the most probable as turbidity increases noticeably during C3 DWRs that coincide with neap tide cycles. The similarity in the timing and physical character of turbidity increases during C3 and those in C1 and C2 suggests that turbidity pulses mark the onset of weak DWRs during October and March, but where the chemical characteristics of DWRs are close to those of the central SoG. Regardless of the driving mechanism, the weak, intermittent DWRs during C3 suggests that the number of DWRs annually might be even higher than those documented in this study and this would increase the annual sedimentation rate estimated in this study.

### Physical DWR characteristics

DWRs initiate during neap tides and at or close to the end of strong flood tide currents. Chemical characteristics typically show rapid changes compared to ambient values at the start of these events while turbidity progressively increases over the course of them (Fig. 3). Although we do not have enough evidence to define the source of increased suspended sediments during DWRs two hypotheses may explain it. 1) Mixing of waters contributes to re-suspension of sediments at Boundary Pass, such that DWRs are already rich in suspended sediments before flowing into SoG. 2) Elevated suspended sediment concentration originates from re-suspension of previously deposited sediments after the DWRs pass Boundary Pass where downslope moving DWRs re-suspend seafloor sediments^21^. It is also possible that these two mechanisms both contribute to turbidity increases in DWRs. For example, mixing can form a high-fluid content sediment layer at the bottom of dense waters and these sediments can re-suspend as the dense waters start flowing in to SoG triggering gravity flows^24^.

The calculated sedimentation rate during each DWR in 2012 was 1.5 cm. Given that 6–15 DWRs occur every year (Supplementary Fig. S1), the annual sedimentation rate can, theoretically, exceed 9 cm yr^−1^ and may reach up to 22.5 cm yr^−1^. Over 11 years, these values would suggest that DWRs deposited approximately 2 m of sediment in the SoG. This value is significantly higher than the reported maximum sedimentation rate in the Fraser prodelta (~3 cm yr-1^25^) suggesting that either sedimentation rates are significantly higher along the pathway of the DWRs (base of the SoG) or that some DWRs and bottom currents contribute to seafloor erosion and reduce the long-term sedimentation rate. Further sampling and monitoring is needed to accurately assess long-term sedimentation rates at the base of the SoG.

In summary, we demonstrate episodic long-lived fluctuations in the physical and chemical ocean characteristics of the Strait of Georgia, Canada showing strong evidence of deep water renewal events (DWRs). Several factors control the timing and character of the DWRs including Pacific Ocean upwelling, Fraser River discharge, El-Nino cycles, and tidal currents^9,13,26^. We document at least six DWRs occur every year refreshing bottom waters in the Strait of Georgia. DWRs are divided into three cycles (C1–C3). C1 and C2 are characterized by pronounced fluctuations in seawater characteristics and are consistent with previously defined DWRs. C3 events display weak fluctuations in chemical parameters and are documented here for the first time. Regardless of the cycle, DWRs initiate during neap tides and are associated with significant increases in turbidity.

For the first time in the literature, DWRs are documented using video footage. Video footage of DWRs in 2012 provides insight into how these events transport and deposit sediments. Available data suggest 1.5 cm of sediment is deposited during each DWR in C1 and C2. With a sedimentation rate of potentially 9 cm yr^−1^, DWRs should be considered an important sediment transport mechanism in strait and enclosed seaways (e.g., fjords).

## Methods

### Data collection

We utilized an extensive dataset of real-time physical and chemical ocean measurements and seafloor video footage. Oceanographic data is derived from instruments situated at 300 m water depth (Fig. 1). All instruments are situated between 1 and 2 m above seafloor. The instrument station is operated and maintained by Ocean Networks Canada (ONC) who supports a network of recording stations on the seafloor on the west coast of Canada. Oceanographic data used in this study include salinity, dissolved oxygen (DO), temperature, turbidity, and acoustic doppler current profiler (ADCP) measurements. Supplementary Table S2 displays the instrument models, sensors and sampling intervals. More detailed information can be found in ONC webpage (https://www.oceannetworks.ca/). Average, minimum and maximum values for each oceanographic property are calculated from time-averaged data.

Video footage used herein was originally collected for a forensic investigation to observe and measure decomposition rates of pigs^27^. Consequently, video footage contains pig bones that are used to estimate sedimentation rates during DWRs. Pig carcasses were deployed in a cage and the bottom of the cage has a mesh size of 1 cm squares. The video camera was staged approximately 1 m above the seafloor and its rotation was controlled by ONC personnel. The camera was activated periodically and recorded 3–4 minute long videos each time. Consequently, video footage of DWRs are intermittent. The original video file is HDWebcam (.asf file format), but we converted the file into Quick Time Movie (.mov file format) to reduce the file size.

The beginning of the DWRs are picked by sudden spikes (positive or negative), particularly in the chemical characteristics, compared to ambient values while the end of DWRs are picked when all the characteristics returned back to ambient values. When describing chemical changes, we compared DWRs with ambient values. We note that DWRs predominantly start at the end of a strong flood tide current, thus ambient values are defined at the end of the previous flood tide.

## Supplementary information

Supplementary Figures

Supplementary Table S1

Supplementary Table S2

Supplementary Video S1

## Data Availability

The physical and chemical real-time oceanographic data used in this study is publicly available from Ocean Networks Canada (http://www.oceannetworks.ca).
