# Supplementary material for: Deep-Water Renewal Events; Insights into Deep Water Sediment Transport Mechanisms
Source: Sci Rep. 2020 Apr 9;10:6139. doi: 10.1038/s41598-020-63123-3 (PMC7145800; doi:10.1038/s41598-020-63123-3)
Supplement: Supplementary file 1 — Supplementary Figures [file 41598_2020_63123_MOESM1_ESM.pdf]

# Deep-Water Renewal Events; Insights into Deep Water Sediment Transport Mechanisms

**K. Ayranci[1]* and S. E. Dashtgard[2]**

[1]College of Petroleum Engineering & Geosciences, King Fahd University of Petroleum & Minerals, Dhahran 31261, Saudi Arabia

[2]Applied Research in Ichnology and Sedimentology (ARISE) Group, Department of Earth Sciences, Simon Fraser University, 8888 University Drive, Burnaby, British Columbia, Canada, V5A 1S6

Corresponding author: Korhan Ayranci (ayranci@gmail.com; korhan.ayranci@kfupm.edu.sa)

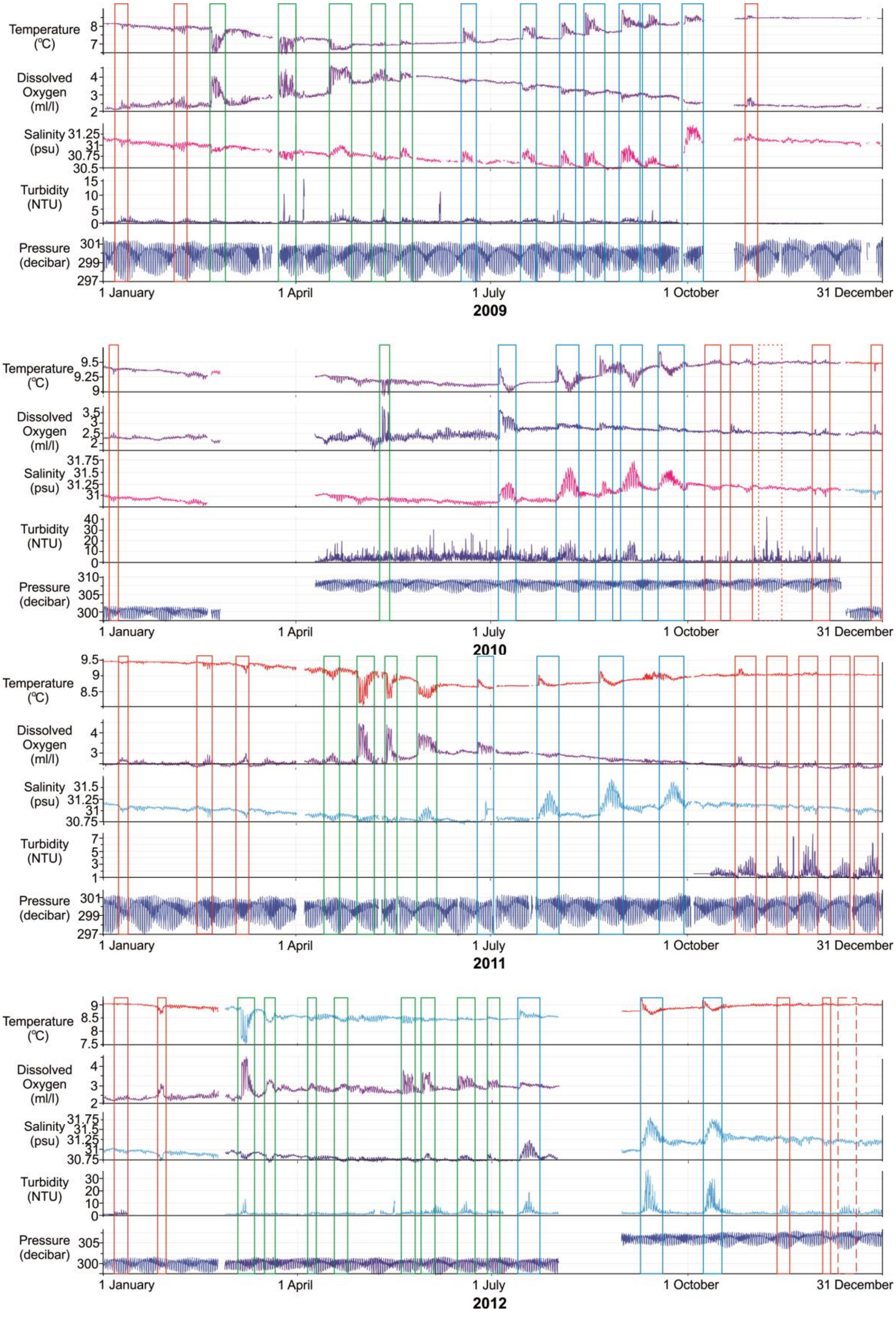
Temperature (°C)
Dissolved Oxygen (ml/l)
Salinity (psu)
Turbidity (NTU)
Pressure (decibar)
1 January
1 April
1 July
1 October
31 December
2009
2010
2011
2012

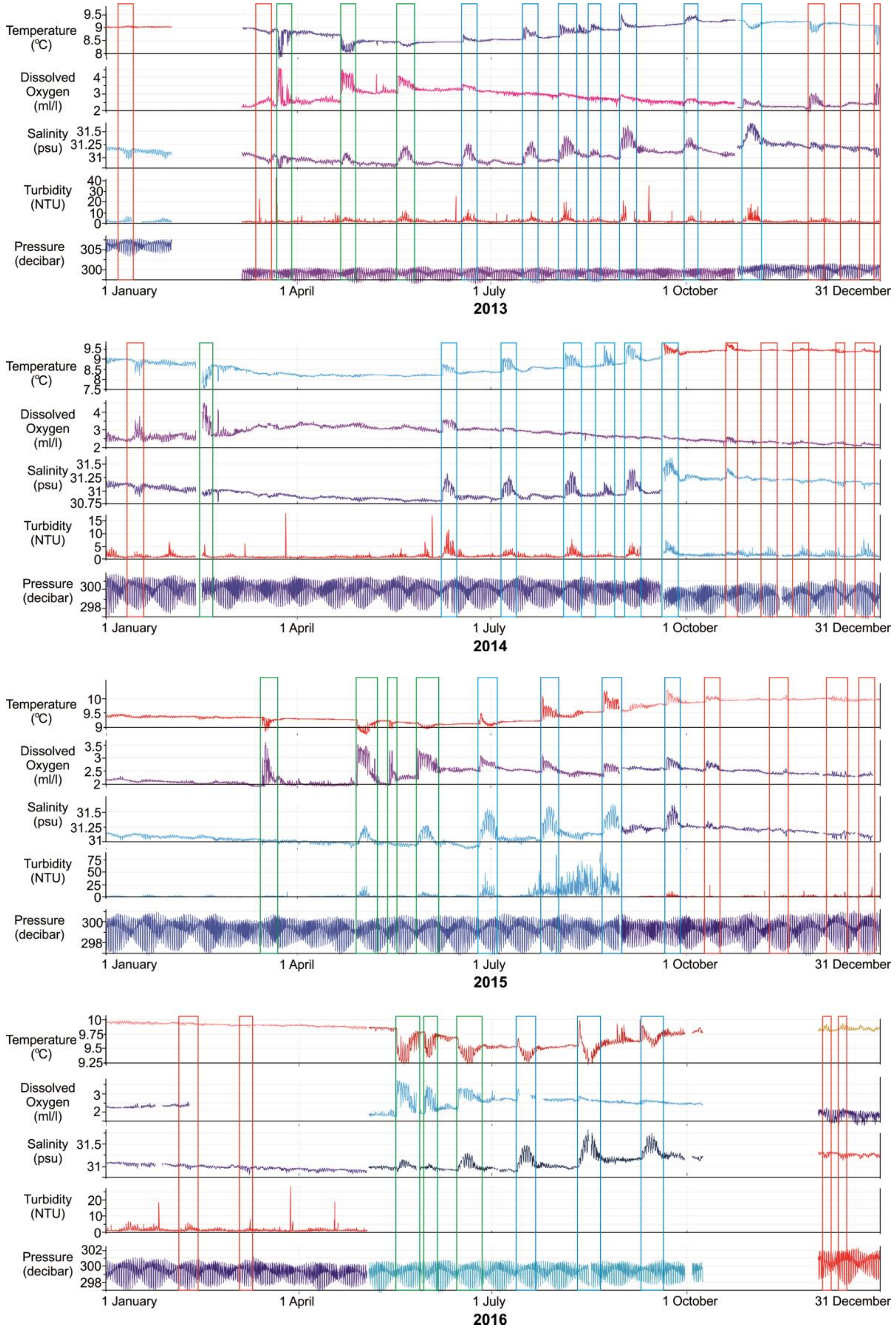
Temperature (°C)
Dissolved Oxygen (ml/l)
Salinity (psu)
Turbidity (NTU)
Pressure (decibar)
1 January
1 April
1 July
1 October
31 December
2013
2014
2015
2016

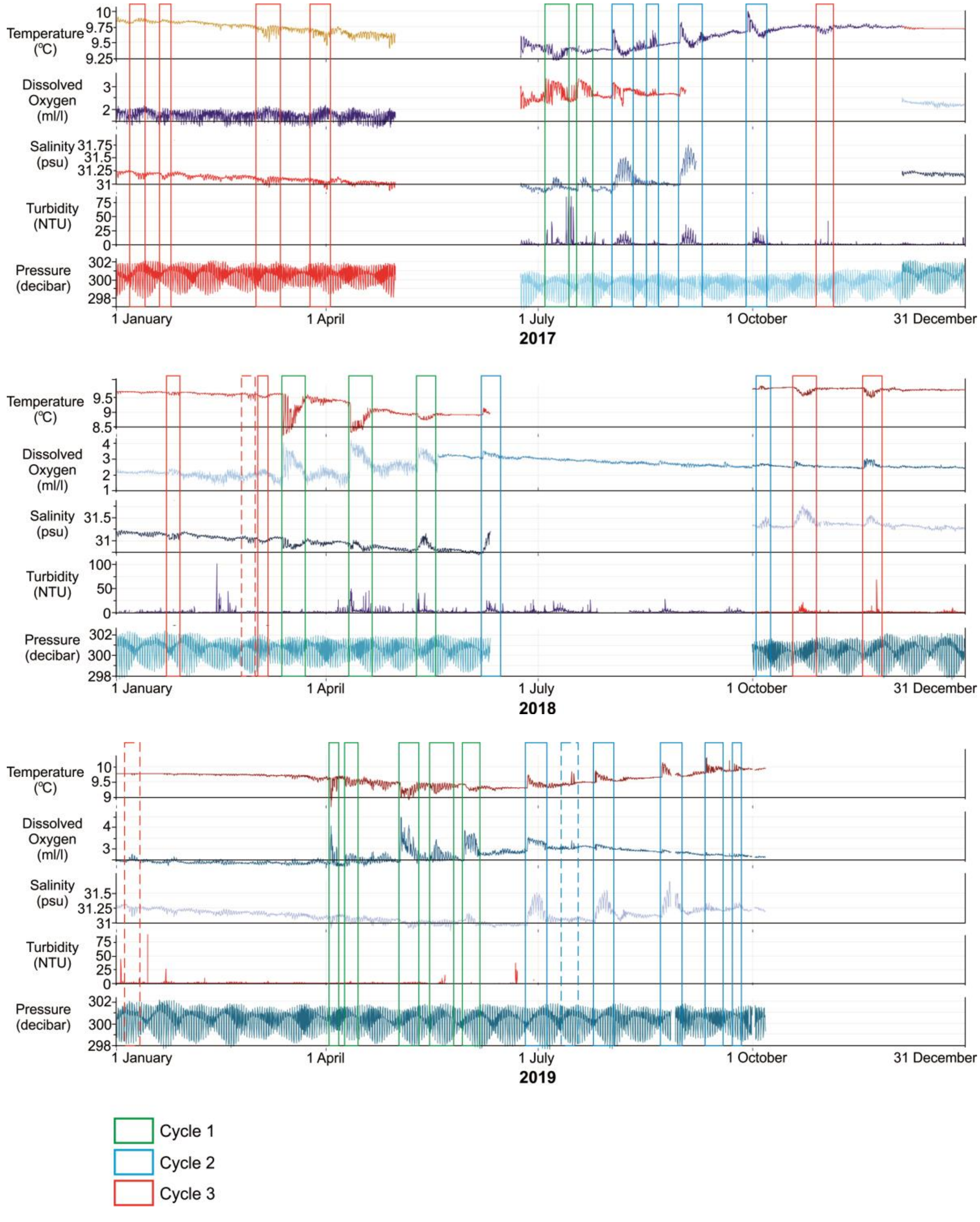


**Supplementary Figure S1.** Eleven years of annual chemical properties of the central Strait of Georgia bottom waters (300 m) between 2009 and 2019. Please note that color change in each graph represent different measuring equipment.

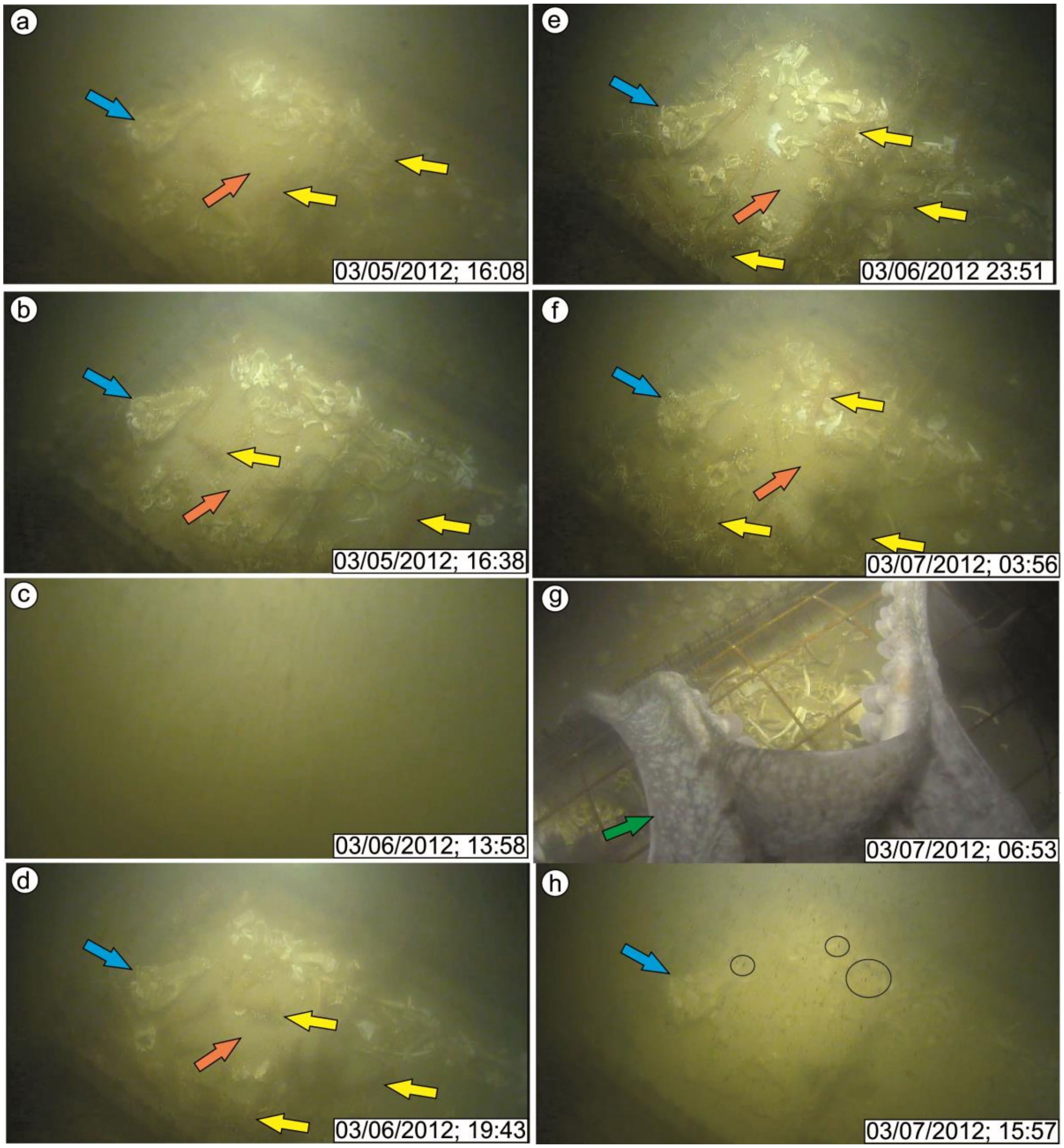


**Supplementary Figure S2.** Additional video captures during the DWR0312 event. In all images, the blue arrow indicates the pig skull, the orange arrow indicates the mesh bottom of the cage, the yellow arrows point to shrimps, and the green arrow points to giant octopus.

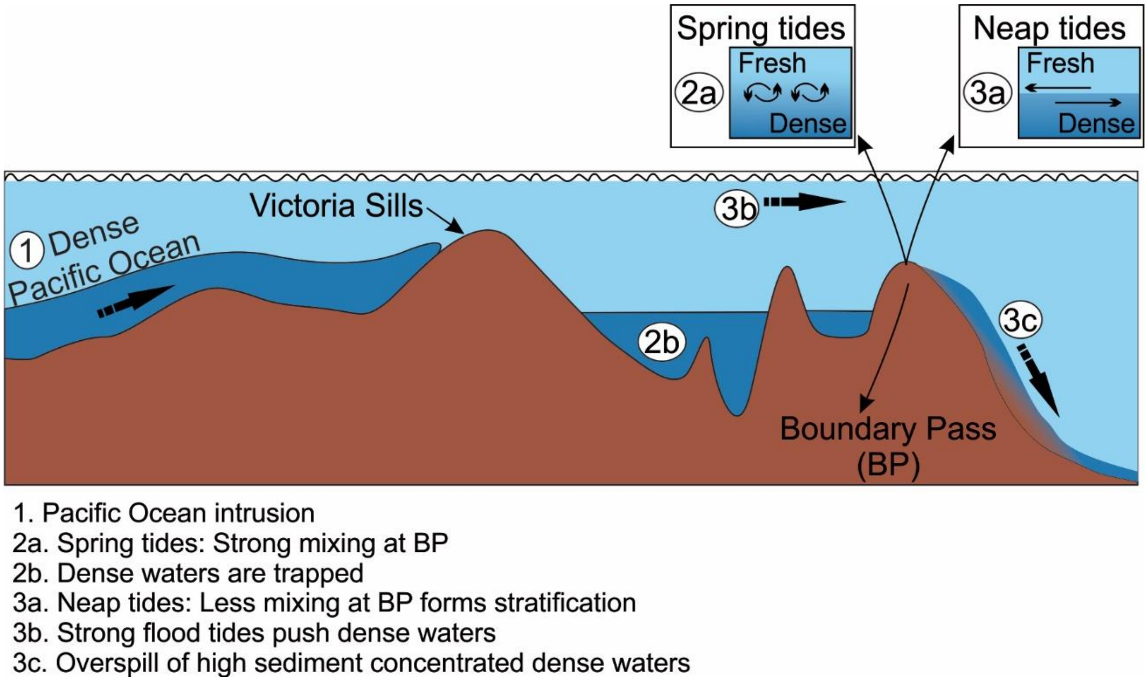


**Supplementary Figure S3.** Schematic illustration of upwelling events trapped by sills in the Salish Sea[1], and their overspilling into the Strait of Georgia.

**Supplementary Video S1.** Video footage covering the second major turbidity peak during the DWR0312 event.

## References

1. Johannessen SC, Macdonald RW. Effects of local and global change on an inland sea: the Strait of Georgia, British Columbia, Canada. *Climate Research* **40**, 1-21 (2009).
