# Supplementary material for: Deep-Water Renewal Events; Insights into Deep Water Sediment Transport Mechanisms
Source: Sci Rep. 2020 Apr 9;10:6139. doi: 10.1038/s41598-020-63123-3 (PMC7145800; doi:10.1038/s41598-020-63123-3)
Supplement: Supplementary file 3 — Supplementary Table S2 [file 41598_2020_63123_MOESM3_ESM.docx]

Detailed information about instruments used in this study.

| **Year** | **Device Name** | **Sensor Type** | **Unit** | **Sampling interval (sec.)** |
| --- | --- | --- | --- | --- |
| **2009** | Sea-Bird SeaCAT SBE16plus 5047 | Temperature | °C | 60-300 |
|  |  | Practical salinity | Psu |  |
|  |  | Pressure | Decibar |  |
|  | Aanderaa Optode 4175 (S/N 0581) | Oxygen Concentration Corrected | ml/l | 60 |
|  | WET Labs ECO-NTUS 117 | Turbidity | NTU | 1-600 |
| **2010** | Sea-Bird SeaCAT SBE16plus 4686, 4996, and 5047 | Temperature | °C | 60-600 |
|  |  | Practical salinity | Psu |  |
|  |  | Pressure | Decibar |  |
|  | Aanderaa Optode 4175 (S/N 0581) and 4175 (S/N 0580) | Oxygen Concentration Corrected | ml/l | 1-600 |
|  | WET Labs ECO-NTUS 117 | Turbidity | NTU | 1-600 |
| **2011** | Sea-Bird SeaCAT SBE16plus 4686 | Temperature | °C | 60-600 |
|  |  | Practical salinity | Psu |  |
|  |  | Pressure | Decibar |  |
|  | Aanderaa Optode 4175 (S/N 0581) | Oxygen Concentration Corrected | ml/l | 60 |
|  | WET Labs ECO-NTUS 117 | Turbidity | NTU | 1-600 |
| **2012** | Sea-Bird SeaCAT SBE16plus 4686 and 6936 | Temperature | °C | 60-600 |
|  |  | Practical salinity | Psu |  |
|  |  | Pressure | Decibar |  |
|  | Aanderaa Optode 4175 (S/N 0581) | Oxygen Concentration Corrected | ml/l | 60 |
|  | WET Labs ECO-NTUS 117 and 319 | Turbidity | NTU | 1-600 |
|  | RDI Workhorse Quartermaster ADCP 150 kHz (SN 8497) | Acoustic doppler current profiler, facing upward |  | 2-30 |
| **2013** | Sea-Bird SeaCAT SBE16plus 4686 and 6936 | Temperature | °C | 60-600 |
|  |  | Practical salinity | Psu |  |
|  |  | Pressure | Decibar |  |
|  | Aanderaa Optode 4175 (S/N 0581) and 4175 (S/N 18) | Oxygen Concentration Corrected | ml/l | 1-60 |
|  | WET Labs ECO-NTUS 319 and 371 | Turbidity | NTU | 1-600 |
| **2014** | Sea-Bird SeaCAT SBE16plus 4686 and 6936 | Temperature | °C | 60-600 |
|  |  | Practical salinity | Psu |  |
|  |  | Pressure | Decibar |  |
|  | Aanderaa Optode 4175 (S/N 0581) | Oxygen Concentration Corrected | ml/l | 60 |
|  | WET Labs ECO-NTUS 319 and 371 | Turbidity | NTU | 1-600 |
| **Year** | **Device Name** | **Sensor Type** | **Unit** | **Sampling interval (sec.)** |
| **2015** | Sea-Bird SeaCAT SBE19plus V2 4997 and Sea-Bird SeaCAT SBE16plus 4686 | Temperature | °C | 1-600 |
|  |  | Practical salinity | Psu |  |
|  |  | Pressure | Decibar |  |
|  | Aanderaa Optode 4175 (S/N 0580) and 4175 (S/N 0581) | Oxygen Concentration Corrected | ml/l | 1-600 |
|  | WET Labs ECO-NTUS 319 and 371 | Turbidity | NTU | 1-600 |
| **2016** | Sea-Bird SeaCAT SBE19plus V2 4997, 7286 and  5270 | Temperature | °C | 0.25-600 |
|  |  | Practical salinity | Psu |  |
|  |  | Pressure | Decibar |  |
|  | Aanderaa Optode 4175 (S/N 0580), 4175C (S/N 1685), and SeaBird 43 Oxygen (S/N 2584) | Oxygen Concentration Corrected | ml/l | 0.25-600 |
|  | WET Labs ECO-NTUS 371 | Turbidity | NTU | 1-60 |
| **2017** | Sea-Bird SeaCAT SBE19plus V2 7286, 5270, and 7793 | Temperature | °C | 0.25-600 |
|  |  | Practical salinity | Psu |  |
|  |  | Pressure | Decibar |  |
|  | Sea-Bird SBE 63 Dissolved Oxygen Sensor 630111, 631533, 630834, 630111, 631533, and SeaBird 43 Oxygen (S/N 2584) | Oxygen Concentration Corrected | ml/l | 0.25-600 |
|  | WET Labs ECO-NTUS 372 | Turbidity | NTU | 1-60 |
| **2018** | Sea-Bird SeaCAT SBE19plus V2 6937 and 7286 | Temperature | °C | 0.25-600 |
|  |  | Practical salinity | Psu |  |
|  |  | Pressure | Decibar |  |
|  | Sea-Bird SBE 63 Dissolved Oxygen Sensor 630111, 631533, and 630834 | Oxygen Concentration Corrected | ml/l | 0.25-600 |
|  | WET Labs ECO-NTUS 371 and 372 | Turbidity | NTU | 1-60 |
| **2019** | Sea-Bird SeaCAT SBE19plus V2 6937 | Temperature | °C | 0.25-60 |
|  |  | Practical salinity | Psu |  |
|  |  | Pressure | Decibar |  |
|  | Sea-Bird SBE 63 Dissolved Oxygen Sensor 630111 | Oxygen Concentration Corrected | ml/l | 1-600 |
|  | WET Labs ECO-NTUS 371 | Turbidity | NTU | 1-60 |
